# 2023 European Society of Cardiology guidelines for the management of cardiovascular disease in patients with diabetes: Statement of endorsement by the NVVC

**DOI:** 10.1007/s12471-025-01967-y

**Published:** 2025-07-09

**Authors:** Fabrice M. A. C. Martens, Frank L. J. Visseren, Jan Westerink, Ruud F. Spee, El Messaoudi Saloua, Arend Mosterd, Louis M. Handoko, Maarten J. G. Leening, Tjeerd J. Römer, Andries Slootweg, Arianne C. van Bon, Martin E. W. Hemels

**Affiliations:** 1 Department of Cardiology, Amsterdam UMC, Amsterdam, The Netherlands; 2 Department of Vascular Medicine, Endocrinology & Diabetes, UMC Utrecht, Utrecht, The Netherlands; 3 Department of Internal Medicine, Isala Hospital, Zwolle, The Netherlands; 4 Department of Cardiology, Maxima Medical Centre, Veldhoven, The Netherlands; 5 Department of Cardiology, Radboud UMC, Nijmegen, The Netherlands; 6 Department of Cardiology, Meander Medical Centre, Amersfoort, The Netherlands; 7 Department of Cardiology, UMC Utrecht, Utrecht, The Netherlands; 8 Department of Cardiology, Epidemiology, and Radiology, Erasmus MC—University Medical Centre Rotterdam, Rotterdam, The Netherlands; 9 Department of Cardiology, Alrijne Hospital, Leiderdorp-Leiden, The Netherlands; 10 Department of Cardiology, Hospital Group Twente, Almelo-Hengelo, The Netherlands; 11 Department of Internal Medicine, Rijnstate Hospital, Arnhem, The Netherlands; 12 Department of Cardiology, Rijnstate Hospital, Arnhem, The Netherlands

**Keywords:** Cardiovascular disease, Diabetes, SGLT2 inhibitors, GLP‑1 receptor agonists

## Abstract

The 2023 update of the European Society of Cardiology guidelines for the management of cardiovascular disease (CVD) in patients with diabetes are designed to guide prevention, early diagnosis, and management of CVD in patients with diabetes and provide recommendations on CVD risk stratification, as well as on screening. This article provides a summary of the key recommendations and a practical approach for cardiologists in the Netherlands to implement these guidelines in daily clinical practice by focusing on recommendations related to type 2 diabetes, including a step-by-step scheme for prescription of Sodium-Glucose Transport Protein 2 Inhibitors (SGLT2) inhibitors and Glucagon-like peptide‑1 (GLP-1) receptor agonists. These agents can be prescribed in addition to standard care, and independent of glucose control, target HbA1c, or obesity.

## Introduction

The 2023 update of the European Society of Cardiology (ESC) guidelines for the management of cardiovascular disease (CVD) in patients with diabetes aims to guide prevention and management of the manifestations of CVD in patients with diabetes [1]. In contrast to the previous 2019 ESC guidelines on diabetes, pre-diabetes, and CVD, less emphasis is put on pre-diabetes given the absence of evidence to facilitate clear treatment recommendations. The new 2023 ESC guidelines mainly focus on two aspects [1, 2, 4]: 1. Screening and cardiovascular risk assessment among patients with diabetes; 2. Evidence-based, personalised treatment strategies in patients with established CVD and type 2 diabetes (T2DM).

For this endorsement statement, we decided to focus on recommendations to type 2 Diabetes Mellitus (T2DM). Specific considerations have been given to the interdisciplinary approach, which should involve healthcare providers from different disciplines and areas of expertise to support shared decision-making and implement a personalised treatment strategy. For other aspects concerning the management of patients with diabetes, we refer to the recommendations from dedicated diabetes associations, e.g., the European Association for the Study of Diabetes or the American Diabetes Association [3].

In this review, practical suggestions towards clinical implementation are made for the specific context of the Netherlands, for targets also based on the 2024 Dutch Multidisciplinary Guidelines for Cardiovascular Risk Management (CVRM), [5] and prevailing health insurance reimbursement criteria for Sodium-Glucose Transport Protein 2 Inhibitors (SGLT2) inhibitors and Glucagon-like peptide‑1 (GLP-1) receptor agonists in the Netherlands. Accordingly, a schematic use of SGLT2 inhibitors and GLP‑1 receptor agonists by cardiologists in clinical practice in the Netherlands is provided.

## Screening and cardiovascular risk assessment (Infobox 1)

### Screening for T2DM in patients with CVD

Given the high prevalence of undetected T2DM, it is recommended that all patients with CVD be screened for the presence of T2DM using fasting plasma glucose levels. After an elevated fasting plasma glucose value of ≥ 7.0 mmol/L, twice on different days, or a random non-fasting glucose ≥ 11 mmol/L, the diagnosis of diabetes is established. Although not supported by current Dutch guidelines, an HbA1c ≥ 48 mmol/mol can also be used to diagnose diabetes.

### Screening for CVD, heart failure and chronic kidney disease in patients with T2DM

Screen known T2DM patients for the presence of CVD, signs and symptoms of heart failure (HF), and chronic kidney disease (CKD).

Calculate 10-year CVD risk scores with SCORE2-Diabetes or lifetime risk & treatment effect with DIAL2-model in patients with diabetes, with and without CVD.

Patients with T2DM are already at increased risk of developing both CVD and CKD. This risk of (new) cardiovascular events increases substantially in the presence of established CVD or impaired (estimated) glomerular filtration rate (eGFR), with and without proteinuria.

**Fig. 1:**
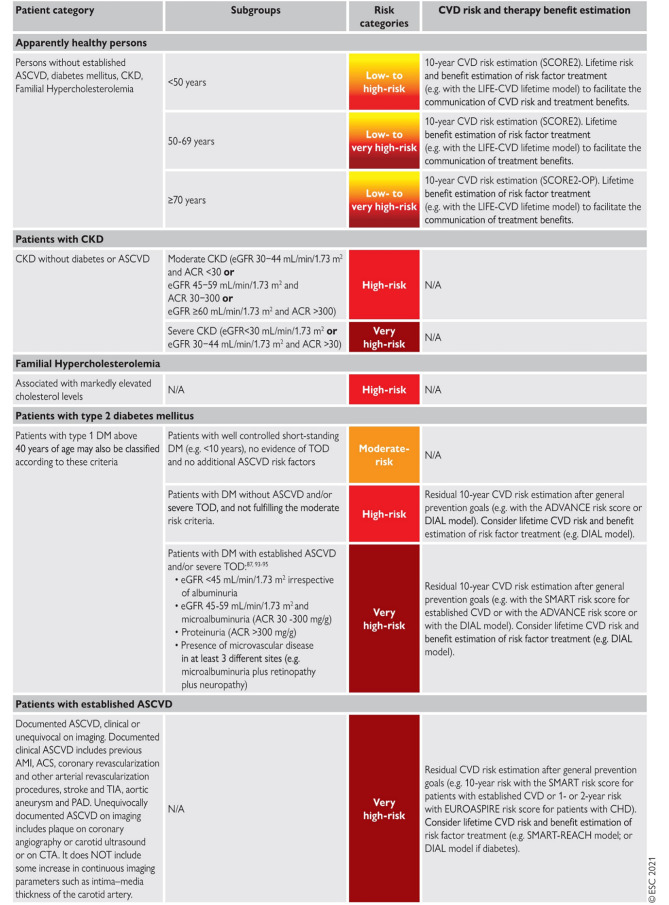
CV-risk categories, adapted from the ESC-guidelines Prevention 2021 and adopted in the Dutch Guidelines CVRM 2024. With permission from Oxford University Press

According to the 2021 ESC CVD Prevention Guidelines [2] and the 2024 Dutch Multidisciplinary Guidelines for Cardiovascular Risk Management (CVRM) [5], CVD risk assessment in patients with T2DM is based on the presence of established CVD or target organ damage, and categorised in three groups (Fig. 1; [5]).

1. ‘Moderate’ CVD risk is defined as patients with T2DM who do not fulfill the ‘very high risk’ criteria and a 10-year CVD risk 5 to 10% using the recommended SCORE2-Diabetes calculator [1, 2, 5].

In the 2024 Dutch Multidisciplinary Guidelines for CVRM, ‘moderate’ CVD risk is also defined as well-regulated diabetes diagnosed less than 10 years ago without target organ damage or other CVD risk factors.

2. ‘High’ CVD risk is defined as patients with T2DM who do not fulfill the ‘very high’ CVD risk criteria and a 10-year CVD risk of 10 to 20% using the recommended SCORE2-Diabetes calculator [1, 5, 6].

CVD risk is also considered ‘high’ if moderate CKD without T2DM is present as defined by:

- an eGFR 30–44 mL/min/1.73 m^2^ and urine albumin-to-creatinine ratio (UACR) < 3 mg/mmol, or
- an eGFR 45–59 mL/min/1.73 m^2^ and UACR 3–30 mg/mmol, or
- an eGFR ≥ 60 mL/min/1.73 m^2^ and UACR > 30 mg/mmol [2, 5]

In the 2024 Dutch Guidelines CVRM, ‘high’ CVD risk is also defined as having diabetes for more than 10 years or not well-regulated but without any target organ damage or established CVD [5].

3. ‘Very high’ CVD risk is defined as patients with T2DM with clinically established CVD, target organ damage, or a 10-year CVD risk ≥ 20% using the recommended SCORE2-Diabetes calculator.

Patients with T2DM are at very high CVD risk due to severe CKD, defined as:

- an eGFR < 45 mL/min/1.73 m^2^ irrespective of albuminuria, or
- an eGFR 45–59 mL/min/1.73 m^2^ and microalbuminuria (UACR 30–300 mg/g), proteinuria (UACR > 300 mg/g), or presence of microvascular disease at ≥ 3 sites (e.g. microalbuminuria, retinopathy, and neuropathy) [1, 2, 5, 6].

CKD without T2DM is considered to convey ‘very high risk’ if:

- an eGFR < 29 mL/min/1.73 m^2^, or
- an eGFR 30–44 mL/min/1.73 m^2^ with microalbuminuria (UACR 3–30 mg/mmol), or
- an eGFR 45–59 mL/min/1.73 m^2^ and UACR > 30 mg/mmol [1, 2, 5, 6].

**Fig. 2:**
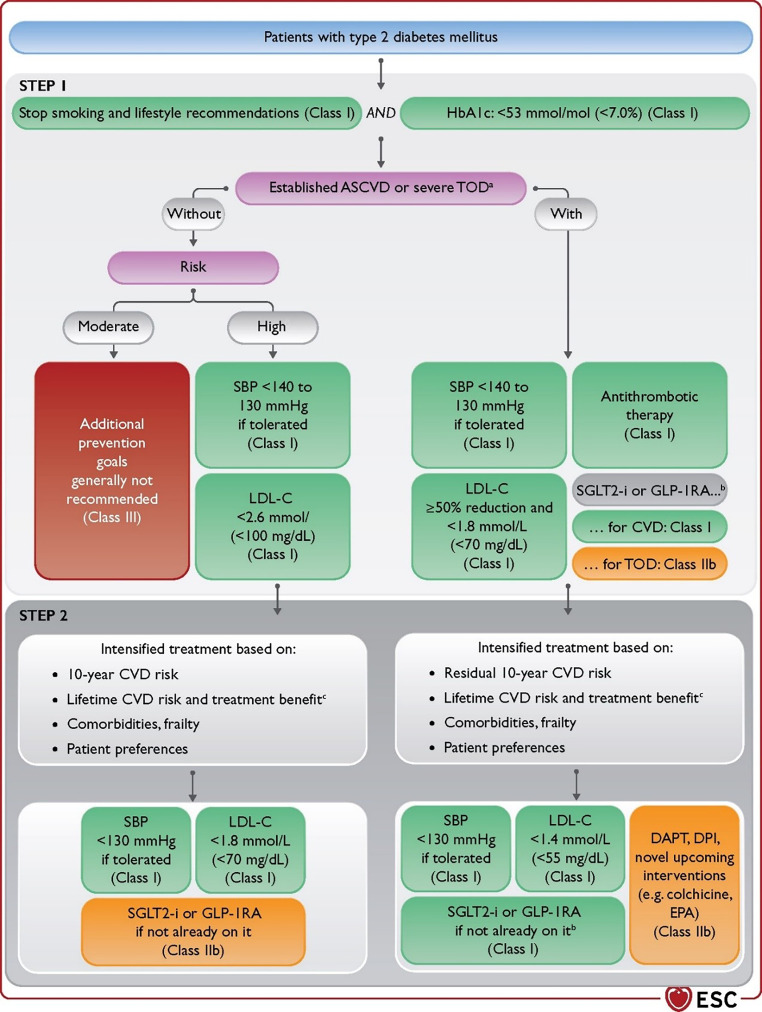
Flow chart of cardiovascular risk and risk factor treatment in patients with type 2 diabetes mellitus. Ultimate treatment goals for SBP (< 130 mm Hg) and LDL‑C (according to level of risk) according to the respective ESC Guidelines [3, 4] are to be pursued as indicated. The stepwise approach has to be applied as a whole: after STEP 1, considering proceeding to the intensified goals of STEP 2 is mandatory. Risk scores are available in the ESC CVD Risk Calculator app for mobile devices (https://www.escardio.org/Education/ESC-Prevention-of-CVD-Programme/Risk-assessment/esc-cvd-risk-calculation-app) and at websites such as https://www.u-prevent.com. *ACR* albumin-to-creatinine ratio, *ASCVD* atherosclerotic cardiovascular disease, *CKD* chronic kidney disease, *CVD* cardiovascular disease, *DAPT* dual antiplatelet therapy, *DM* diabetes mellitus, *eGFR* estimated glomerular filtration rate, *ESC* European Society of Cardiology, *GLP-1RA* glucagon-like peptide‑1 receptor agonist, *HbA1c* glycated haemoglobin, *HF* heart failure, *LDL‑C* low-density lipoprotein cholesterol, *SBP* systolic blood pressure, *SGLT2* sodium-glucose cotransporter 2, *TOD* target organ damage (retinopathy, nephropathy, neuropathy). ^a^Severe TOD is defined as at least one of: eGFR < 45 mL/min/1.73 m^2^ irrespective of the presence or absence of albuminuria; eGFR 46–59 mL/min/1.73 m^2^ and microalbuminuria (ACR 30–300 mg/g or 3–30 mg/mmol); proteinuria (ACR > 300 mg/g or > 30 mg/mmol); presence of microvascular disease in at least three different sites (e.g. microalbuminuria plus retinopathy plus neuropathy). ^b^Patients with prevalent HF or CKD are recommended for SGLT2 inhibitor, and patients post stroke are recommended for GLP-1RA treatment. ^c^Lifetime treatment benefit is expressed as extra CVD-free life gained from a certain intervention or treatment intensification. With permission from Oxford University Press

**Fig. 3:**
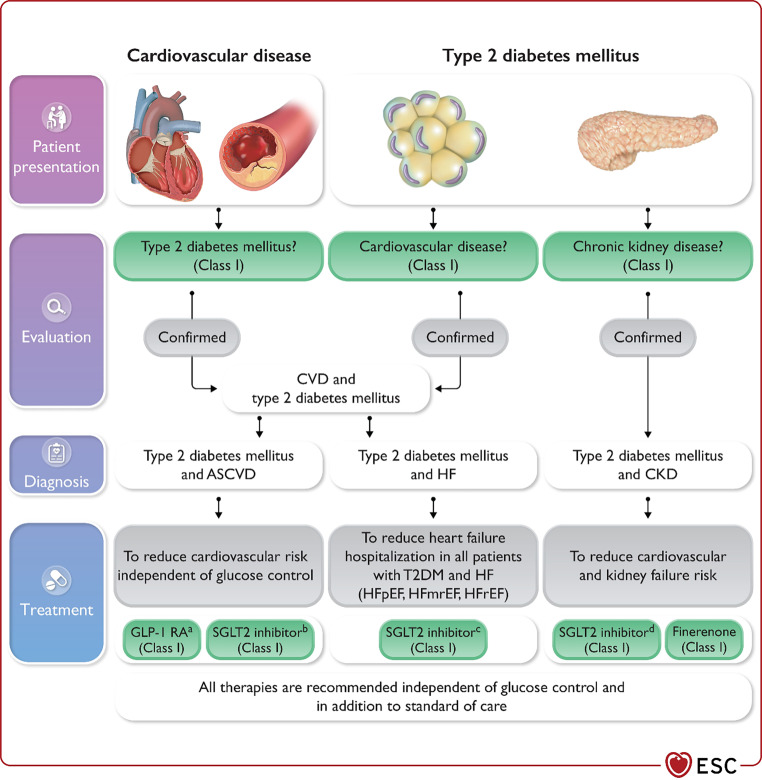
Management of cardiovascular disease in patients with type 2 diabetes: clinical approach and key recommendations. *ASCVD* atherosclerotic cardiovascular disease, *CKD* chronic kidney disease, *CVD* cardiovascular disease, *GLP‑1 RA* glucagon-like peptide‑1 receptor agonist, *HF* heart failure, *HFmrEF* heart failure with mildly reduced ejection fraction, *HFpEF* heart failure with preserved ejection fraction, *HFrEF* heart failure with reduced ejection fraction, *s.c.* subcutaneous, *SGLT2* sodium-glucose co-transporter‑2, *T2DM* type 2 diabetes mellitus. ^a^GLP‑1 RAs with proven cardiovascular benefit: liraglutide, semaglutide s.c., dulaglutide, efpeglenatide. ^b^SGLT2 inhibitors with proven cardiovascular benefit: empagliflozin, canagliflozin, dapagliflozin, sotagliflozin. ^c^Empagliflozin, dapagliflozin, sotagliflozin in HFrEF; empagliflozin, dapagliflozin in HFpEF and HFmrEF. ^d^Canagliflozin, empagliflozin, dapagliflozin. With permission from Oxford University Press

Both CVD and CKD have a major impact on prognosis and subsequent preventive treatment strategies in patients with T2DM. Therefore, it is important to identify the co-existence of T2DM and CVD or CKD on an annual basis (Figs. 2 and 3; [1, 2]) CKD should be screened for by assessing eGFR, defined by the CKD-EPI formula as well as UACR.

Furthermore, opportunistic screening including a systematic survey for HF symptoms and/or signs of HF is also recommended at outpatient visits or during clinical encounters in all patients with diabetes.

#### Infobox 1

**Screening for T2DM in patients with CVD and screening for CVD in patients with T2DM**

Given the high prevalence of undetected T2DM, it is recommended that all patients with CV disease be screened for the presence of T2DM using fasting plasma glucose levels. Also, screen all patients with T2DM for the presence of CV disease, signs and symptoms of HF, and chronic kidney disease (CKD).—Categorise CV risk in patients with T2DM based on the presence of CV disease and target organ damage, or, in patients aged ≥ 40 years without CV disease or severe target organ damage, based on the results of the SCORE2-Diabetes CV disease risk score.

## Evidence-based, personalised treatment strategies in patients with CVD and T2DM (Infobox 2)

The most important message is to treat CV-risk instead of glycemic control.

Lifestyle modification is recommended as the cornerstone for preventing and managing T2DM to improve glycemic control and more important CVD-risk reduction. In the Netherlands, reimbursement is available for the combined lifestyle intervention (GLI), and specifically for T2DM patients the lifestyle program ‘*Keer Diabetes2 Om’*. Lifestyle modification includes weight reduction, improving diet, increasing physical activity and exercise, as well as smoking cessation. Especially in those with T2DM and obesity, weight reduction is a central goal.

In addition, according to the ESC Guidelines, it is recommended to implement blood pressure control when office blood pressure is ≥ 140/90 mm Hg and aim for a systolic blood pressure of at least < 120 mm Hg at ‘very high’ CVD risk and no frailty. The Dutch Guidelines recommend a target systolic blood pressure of at least < 130 mm Hg for non-frail patients with a ‘very high’ CVD risk.

Moreover, lipid-lowering therapy is a priority for the reduction of CVD risk in patients with diabetes. The ESC Guidelines recommend to aim for an LDL at least < 2.6 mmol/l (< 3.0 mmol/l in the Dutch Guideline), but more specific at least < 1.8 mmol/l (< 2.6 mmol/l in the Dutch Guideline) when at ‘high’ risk for CVD, and < 1.4 mmol/l (< 1.8 mmol/l in the Dutch Guideline for non-frail patients) when having T2DM with already CVD. Statins are recommended as the primary LDL-C-lowering treatment based on the CVD risk profile and the LDL‑C (or non-HDL-C) target levels.

All patients with T2DM and atherosclerotic CVD should receive antithrombotic therapy. Often, a strategy combining antithrombotic therapy, anti-hypertensive drugs and lipid-lowering agents should be implemented (Figs. 2 and 3; [1, 2]) (All Class I indication with Level of evidence A).

### Preferred CVD risk-lowering agents in T2DM

For patients with T2DM, the use of glucose-lowering agents with proven CVD risk reduction should be prioritised, followed by agents with proven CVD safety over those without demonstrated CVD risk reduction or proven CVD safety (Class I indication with Level of evidence C).

The 2023 ESC Guidelines categorise glucose-lowering agents into four groups:

1. Glucose-lowering agents *with proven risk reduction* in randomised clinical trials: SGLT2 inhibitors (empagliflozin, canagliflozin, dapagliflozin, and sotagliflozin) and specific GLP‑1 receptor agonists (liraglutide, subcutaneous semaglutide, dulaglutide, and efpeglenatide);
2. Glucose-lowering agents *with suggested benefit* in randomised clinical trials: metformin and pioglitazone;
3. Glucose-lowering agents *with proven safety* in randomised clinical trials: DPP-4 inhibitors (saxaliptin, sitagliptin, alogliptin, and linagliptin), ertugliflozin, specific sulfonylureas (glimepiride and gliclazide), insulin glargine or insulin degludec, and other GLP‑1 receptor agonists (lixisenatide, exenatide, and oral semaglutide);
4. Glucose-lowering agents *without CVD safety evaluation*: e.g., short-acting insulins and other sulfonylureas.

The prevailing Dutch General Practitioners (GP) guidelines Diabetes mellitus type 2 (NHG Guidelines-Diabetes Mellitus type 2) advise the use of glucose-lowering agents with proven CVD risk reduction in patients with T2DM at ‘very high’ CVD risk [7].

**Fig. 4:**
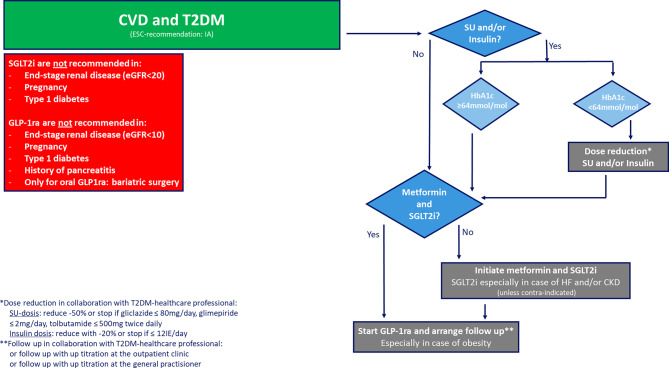
A step by step scheme for prescription of SGLT2 inhibitors and GLP‑1 receptor agonists in patients with CVD and T2DM (irrespective of HF, CKD or obesity but taken current Dutch reimbursement-criteria into account) by cardiologists in clinical practice in the Netherlands

Thus, in order to reduce CVD risk in patients with T2DM with established CVD both GLP‑1 receptor agonists, and SGLT2 inhibitors are recommended, given their proven CVD risk reduction. In the prevailing iteration of the NHG Guidelines-Diabetes Mellitus type 2 an SGLT2 inhibitor is recommended as the first-line treatment, followed by metformine and GLP‑1 receptor agonists in patients at ‘very high’ CVD risk. A step-by-step scheme for prescription SGLT2 inhibitors and GLP‑1 receptor agonists in daily clinical practice is provided in Fig. 4 taking current Dutch reimbursement criteria into account. These agents can be prescribed on top of standard of care including GLI, and independent of glucose control, target HbA1c, or obesity. However, especially in case of HF, SGLT2i is strongly recommended. In case of obesity, treat with GLP‑1 receptor agonists if possible.

The prescription of glucose-lowering agents with suggested CVD risk reduction (i.e. metformin and pioglitazone) is still recommended for patients with T2DM without CV disease or already target organ damage. Glucose-lowering agents with only proven CVD safety, but without proven CVD risk reduction—such as specific sulfonylureas (i.e. glimepiride and gliclazide), or DPP-4 inhibitors (i.e. sitagliptin, alogliptin, linagliptin, and saxagliptin)—should ideally be replaced by SGLT2 inhibitors and GLP‑1 receptor agonists. However, in daily practice, reimbursement criteria must be taken into account.

Glucose-lowering agents without proven CVD safety evaluation should be reserved for situations when other aforementioned options are exhausted. Moreover, treatment strategies should be tailored to avoid hypoglycemias (Fig. 3; [1]).

In patients with HF—irrespective of ejection fraction—it is recommended that all patients with T2DM be treated with an SGLT2 inhibitor in addition to standard care, to reduce HF hospitalisation and CVD death (Class I indication with Level of evidence A). Pioglitazone and saxagliptin are not recommended in patients with T2DM with HF or at increased risk for HF.

**Fig. 5:**
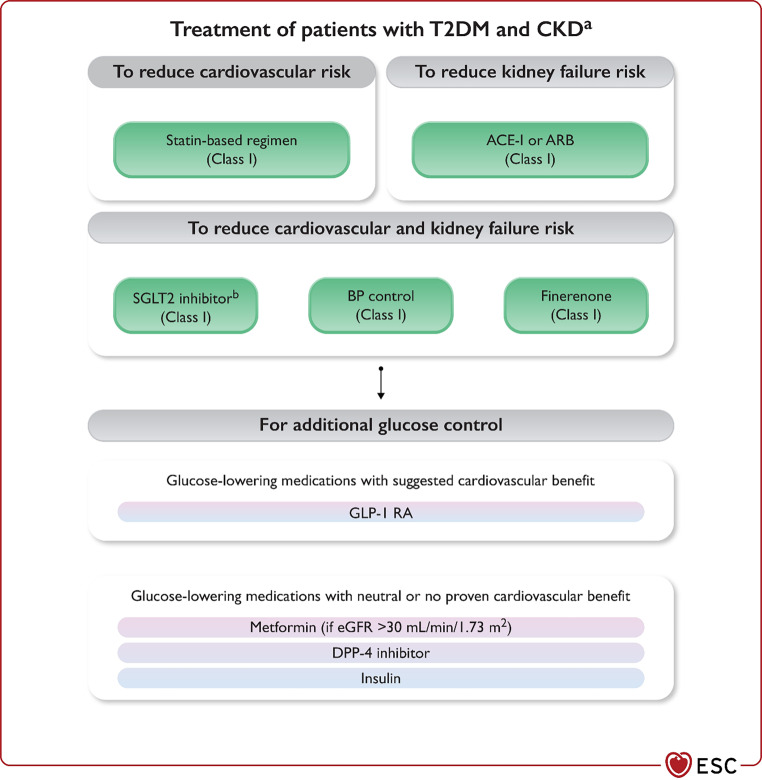
Pharmacological management to reduce cardiovascular or kidney failure risk in patients with type 2 diabetes and chronic kidney disease. *ACE‑I* angiotensin-converting enzyme inhibitor, *ARB* angiotensin-II receptor blocker, *BP* blood pressure, *CKD* chronic kidney disease, *CV* cardiovascular, *CVD* cardiovascular disease, *DPP‑4* dipeptidyl peptidase‑4, *eGFR* estimated glomerular filtration rate, *GLP‑1 RA* glucagon-like peptide‑1 receptor agonist, *RAS* renin–angiotensin system, *SGLT2* sodium–glucose co-transporter‑2, *T2DM* type 2 diabetes mellitus, *UACR* urinary albumin-to-creatinine ratio. ^a^A statin-based regimen reduces CV risk in CKD while ACE‑I or ARBs reduce kidney failure risk; SGLT2 inhibitors, BP control, and finerenone reduce both CV risk and kidney failure risk. SGLT2 inhibitors, RAS inhibitors, and finerenone are particularly effective at reducing risk of kidney failure when albuminuria is present (e.g. UACR ≥ 3 mg/mmol (30 mg/g); stage A2 and A3). ^b^Canagliflozin, empagliflozin, or dapagliflozin. With permission from Oxford University Press

Patients with T2DM and CKD should receive a statin, as well as treatment with angiotensin-converting enzyme inhibitors (ACEi) or angiotensin-II receptor blockers (ARB), and appropriate blood pressure control (ESC-target ≤ 120 mm Hg and Dutch-target at least < 130 mm Hg for non-frail patients). To reduce the risk of both CVD and kidney failure, these patients should be treated with an SGLT2 inhibitor (empagliflozin or dapagliflozin), provided the eGFR ≥ 20 mL/min/1.73 m^2^ at the time of SGLT2 inhibitor prescription. The non-steroidal mineralocorticoid receptor antagonist finerenone is recommended in addition to an ACEi or ARB when:

- an eGFR ≥ 60 mL/min/1.73 m^2^ with a UACR ≥ 30 mg/mmol, or
- an eGFR 25–60 mL/min/1.73 m^2^ and UACR ≥ 3 mg/mmol (All Class I indication with Level of evidence A) (Fig. 5; [1]).

In addition, personalised HbA1c targets are recommended, when possible with a tight target (< 53 mmol/mol) to prevent microvascular complications. HbA1c targets should be adjusted based on the balance between frailty, life expectancy, anticipated benefit, risk of hypoglycemias, and treatment side-effects.

In general, early detection of T2DM and implementation of glucose-lowering treatment with generic (and thereby affordable) agents such as metformin, antihypertensives, and statins has been shown to lower CVD risk in patients with T2DM without manifest CVD.

Finally, management of CVD in patients with T2DM benefits from a multidisciplinary approach (intensive collaboration between all healthcare-professionals involved, especially taken into account the healthcare-professionals who are responsible for the diabetes-care at the GPs) to implement evidence-based personalised strategies to reduce the burden of disease and to improve prognosis.

#### Infobox 2

**Recommendations for personalised treatment strategies in patients with CVD and T2DM in the Netherlands**

Evidence-based recommendations for personalised treatment in T2DM includes:

- Improve lifestyle.
- Implement blood pressure control when office blood pressure is ≥ 140/90 mm Hg aiming for a target of < 120 mm Hg (the Dutch Guideline recommends at least < 130 mm Hg for non-frail patients).
- Prescribe statins or other lipid-lowering drugs based on the CVD risk profile and recommended lipid targets for non-HDL (both LDL and TGs).
- Prioritise the use of glucose-lowering medications with proven CVD risk reduction benefits, followed by agents with proven CV safety, over agents without proven CV benefit or proven CV safety. Especially in case of HF, SGLT2i is mandatory. In case of obesity, treat with GLP‑1 receptor agonists if possible.Treat patients with CVD and T2DM with a statin, an ACEi or ARB, appropriate blood pressure control (≤ 120/80 mm Hg), and antitrombotic therapy.
- Treat patients with T2DM and HF—irrespective of ejection fraction**—**with an SGLT2 inhibitor.
- Treat patients with T2DM and CKD with an SGLT2 inhibitor, a statin, an ACEi or ARB, possibly finerenone, and appropriate blood pressure control.
- Use personalised HbA1c targets, with a possible tighter target (< 53 mmol/mol) to prevent microvascular complications.

## What is new and useful to know for cardiologists?

- Categorise CVD risk in patients with T2DM based on the presence of CVD and target organ damage, or—in patients aged ≥ 40 years without CVD or target organ damage—based on the SCORE2-Diabetes CVD risk score.
- The primary treatment goal is CVD risk reduction, followed by glycemic control in cases of residual dysglycemia. Prioritise the use of glucose-lowering medications with proven CVD risk reduction, followed by agents with proven CVD safety over those without proven CVD risk reduction benefit or safety. SGLT2 inhibitors and GLP‑1 receptor agonists are preferred for treatment of T2DM.
- Treat patients with CVD and T2DM with a statin, an ACEi or ARB, and appropriate blood pressure control (≤ 120 mm Hg; according to the Dutch Guideline at least <130 mm Hg for non-frail patients). In case of obesity, treat with GLP‑1 receptor agonists if possible.
- Treat patients with T2DM and heart failure—irrespective of ejection fraction—with an SGLT2 inhibitor.
- Treat patients with T2DM and CKD with an SGLT2 inhibitor, a statin, an ACEi or ARB, possibly finerenone, and appropriate blood pressure control.
- Use personalised HbA1c targets, with a possible tighter target (< 53 mmol/mol) to prevent microvascular complications.
- Management of CVD in patients with T2DM benefits from a multidisciplinary approach: intensive collaboration between all healthcare-professionals involved to implement evidence-based personalised strategies to reduce the burden of disease and to improve prognosis.

## Deviations from the ESC Guidelines for daily clinical practice in the Netherlands

The recommendations of the 2023 ESC guidelines for the management of CVD in patients with T2DM are largely applicable in the Netherlands. Nonetheless, there are recommendations in the current ESC guideline that are not ideally suited to the specific context of the Netherlands:

- In the NHG Guidelines—Diabetes Mellitus type 2, fasting glucose, and not Hb1Ac, is recommended to diagnose diabetes. Although not supported by prevailing Dutch Guidelines, an HbA1c ≥ 48 mmol/mol can also be used to diagnose diabetes.
- Although the Dutch Guidelines (CVRM and T2DM) have less strict non-HDL and blood pressure targets than several ESC Guidelines to achieve an unequivocal policy of specialists and general practitioners, the Dutch Guidelines (CVRM and T2DM) also make more strict non-HDL and blood pressure targets possible if it seems necessary.
- For patients with (a very high risk for) CVD and T2DM, GLP‑1 receptor agonists are reimbursed in the Netherlands as add-on SGLT2i and metformin, however for T2DM-patients without (a very high risk for) CVD GLP‑1 receptor agonists are only reimbursed if there is a BMI > 30 kg/m^2^ on top of metformin + sulfonylurea derivates or insulin. For obese patients without (a very high risk for) CVD or T2DM, currently only GLP‑1 receptor agonist liraglutide (Saxenda) is reimbursed when a BMI ≥ 35 kg/m^2^ + CVD/OSAS/arthrosis or BMI ≥ 40 kg/m^2^ and at least 1 year Combined Lifestyle Program (GLI) is followed.
- The ESC Guidelines for CVD & DM recommend screening for HF in all patients by means of NT-proBNP testing. However, screening all patients with diabetes using NT-proBNP is not recommended in Dutch Guidelines, due to concerns about diagnostic accuracy and resulting treatment indications [8]. We suggest a more pragmatic approach by actively following up patients with T2DM for signs and symptoms of HF. In cases of diagnostic uncertainty, additional diagnostic testing (inclusive NT-proBNP) should be considered to make or refute a diagnosis of HF.
- Routine assessment of serum iron status to check for iron deficiency is not recommended for all patients with diabetes in the Netherlands, only in the presence of HF or anemia.
- Systematic screening for lower-extremity arterial disease with ankle-brachial index is not recommended in the Netherlands, partly because the detection of asymptomatic abnormal ankle-brachial index does not change treatment. Rather, the specific recommendation would be to use clinical assessment for signs and symptoms.

## Conclusion

The new 2023 ESC guidelines for the management of CVD in patients with diabetes primarily focus on screening, and cardiovascular risk assessment in patients with diabetes, and evidence-based, personalised treatment strategies in patients with established CVD and T2DM.

The recommendations are to screen all patients with T2DM for the presence of CV disease, signs and symptoms of HF, and CKD, and categorise CV risk in patients with T2DM based on the presence of CV disease and target organ damage, or, in patients aged ≥ 40 years without CV disease or severe target organ damage, based on the results of the SCORE2-Diabetes CV disease risk score.

The most important message is to treat CV-risk instead of glycemic control. Therefore, for patients with T2DM, together with lifestyle improvement, optimising blood pressure control and lipid-lowering, the use of glucose-lowering agents with proven CVD risk reduction should be prioritised followed by agents with proven CVD safety over those without proven CVD risk reduction or proven CVD safety.

Specific considerations have been given to the interdisciplinary approach, which should involve healthcare providers from different disciplines and areas of expertise to support shared decision-making and implement a personalised treatment strategy.
